# Pathology image-based predictive model for individual survival time of early-stage lung adenocarcinoma patients

**DOI:** 10.1038/s41598-025-16073-7

**Published:** 2025-10-15

**Authors:** Vi Thi-Tuong Vo, Hyung-Jeong Yang, Taebum Lee, Soo-Hyung Kim

**Affiliations:** 1 Department of Artificial Intelligence Convergence, Chonnam National University, Gwangju, 61186 South Korea; 2 Department of Pathology, Chonnam National University Medical School, Gwangju, 61186 South Korea; 3 Department of Nuclear Medicine, Chonnam National University Hwasun Hospital, Jeollanam-do, South Korea

**Keywords:** Biomedical engineering, Non-small-cell lung cancer

## Abstract

The tumor microenvironment (TME) is associated with tumor prognosis, immunotherapy response, and prognosis in patients. Here, we hypothesized that the entire TME in pathology image is associated with the survival time prediction. To address this hypothesis, we utilize the entire TME on pathology image of early-stage lung adenocarcinoma (esLUAD), which is the most common histological subtype of lung cancer. Notably, we investigated whether machine learning models can predict individual survival time from pathology images without region-level annotation and solely based on patient-level survival data. In particular, we proposed a pathology image-based predictive model in a cascaded learning system to predict the individual survival time of esLUAD patients in two independent cohorts (National Lung Screening Trial (NLST) and Cancer Genome Atlas Program (TCGA)). Besides that, we estimate a mean absolute error (MAE) score and a C-index score that are strongly associated with the survival time prediction. Our method achieved (361.90 MAE - 0.70 C-index) and (365.67 MAE - 0.58 C-index) in early-stage NLST and early-stage TCGA cohorts, respectively. Together, the presented results highlight the importance of computation pathology algorithms in predicting survival time using the entire TME information in pathology images and support the use of computational methods to improve the efficiency of clinical trial studies.

## Introduction

Lung adenocarcinoma (LUAD) is the most prevalent lung cancer, represents about 40-50% of all lung cancer cases. Within lung adenocarcinoma, histology is heterogeneous and associated with various tumor invasion and clinical outcomes^1^.

In addition, invasiveness is one of the hallmarks of cancer and is directly related to metastatic potential and clinical outcomes of the tumor. Although the mortality of patients with lung cancer has decreased in recent years, the mortality rate remains high with an average 5-year survival rate of 15%^2^. With the development of various therapeutic modalities, such as immunotherapy and targeted therapy, surgery, the overall esLUAD has improved approximately 30–50% of early-stage patients, metastases with a 70% and 35% overall 5-year survival rate of stage 1 and stage 2 patients with non-small cell lung cancer, respectively^3,4^.

The increase in survivability is attributed to the implementation of early detection screening programs and the development of targeted therapies and immunotherapies that are effective in specific subtypes of metastatic lung cancer. Therefore, in this study, we present an approach that focuses on therapeutic approaches that target molecular subtypes of early-stage lung adenocarcinoma (esLUAD) and promise to improve outcomes for this important patient group.

**Table 1:** Taxonomy of survival time analysis.

Model Input	Model Output	Time dependency of model output	Example of modeling
Clinical information, CT/PET image, gene expression, protein biomarkers	Survival probability	Time-dependent	Nnet-survival^5^
	Log-risk score	Time-independent	DeepSurv^6^
	Cumulative hazard	Time-dependent	Random survival forest^7^

**Table 2:** Taxonomy of survival time analysis measurements.

Methods	Time dependency	Description
Concordance index	Time-independent	Agreement between the predicted and the observed order of the events
ROC AUC	Time-dependent	Probability of the model to discriminate between the two outcomes
Calibration plot	Time-dependent	Plot of event probability values
Brier score	Time-dependent	Mean squared error of the predicted risks

As an illustration, Table 1 presents the taxonomy of survival time analysis fields, while Table 2 presents the taxonomy of survival time analysis measurements. Accordingly, survival analysis is generally performed^8–13^ using gene expression, protein biomarkers^14–16^, Computed Tomography (CT) or Positron emission tomography (PET) images, along with other clinical or demographic information and patient characteristics such as age, gender, Body Mass Index (BMI), ethnicity, etc. For example, Torkey et al.^17^ proposed a novel deep learning-based method for survival analysis on RNAseq microarray data. Furthermore, the outputs of survival prediction models can be categorized into two types: time-dependent and time-independent^18^. In the time-dependent type, the output value of the prediction model is separately generated for each follow-up time for each patient. In this way, one patient had multiple output values, one for each follow-up time, such as the survival probability or the cumulative hazard function at specified time points. In the time-independent type, the output value of the prediction model is a single value for each patient, regardless of follow-up time. Typically, these output values indicate the overall risk of death in a patient, such as log-risk score.

In recent years, the tissue-based prognosis of the tumor microenvironment (TME) is emerged, consisting of composite structures of normal, malignant cells, connective tissue infiltrated with immune cells, and vessels. Particularly, the prognostic significance of different components of TME in multiple cancers was presented in^19–22^.

Furthermore, hematoxylin and eosin stained images or Whole-slide images (WSIs) are the gold standard for TME analysis. Some studies^23,24^ proposed a pathology-based survival analysis system, based on deep neural network framework and a Cox proportional hazards model with the ground truth of the tumor region. Due to the increase in deep learning and the availability of scanned tissue slides, the pathology-based prognosis is gaining more attention^25^. Wulczyn et al.^26^ proposed the CNN-based weakly supervised learning approach for directly predicting disease specific survival using histopathology images. Hao et al.^27^ proposed a novel patch-wise texture-based convolutional neural network, with a patch aggregation strategy (PAGE-Net) to predict patients survivals using histopathological images and genomic data. Another CNN-based model (Siamese Survival CNN)^28^ reduces histopathology images to whole slide feature maps and uses them with clinical features as input to obtain the survival output. Zhu et al.^24^ proposed a deep convolutional survival model (DeepConvSurv) for survival analysis with pathological images by augmenting image patches. Multiple patches from ROIs were extracted and assigned with patients’ labels. The DeepConvSurv achieved a 0.629 C-Index on the National Lung Screening Trial (NLST) lung cancer data. Katzman et al.^6^ proposed a multi-layer feed forward network (DeepSurv), of which the output is a negative log partial likelihood, in order to provide personalized treatment recommendations. Then Kim et al.^29^ compared the performance of DeepSurv, random survival forest and the Cox proportional hazard model on survival prediction of oral cancer patients. The results of their study suggested that deep learning-based survival prediction may improve prediction accuracy, allowing this method to guide clinicians in better diagnostic and treatment planning.

Despite the overwhelming increase in deep learning-related approaches for histopathology image analysis^26,30–33^, there are several challenges that may hinder the development of these algorithms for routine clinical practice. First, WSIs are often generated with much larger sizes (e.g., 100,000 x 100,000 pixels) than natural medical images (PET/CT and MRI images), thus preventing their initiation as input of a convolutional neural network (CNN). To address this problem, the patch-based method was proposed and a small number of these patches were inputted into the CNN model^34–39^. Especially, InceptionResNetV2^40^^41^ has been applied to pathology image analysis with effective classification results. However, with a large number of parameters (approximately 55.90M), InceptionResNetV2 is not able to significantly increase the training efficiency when the model depth is large. Contrarily, with a small number of parameters (approximately 25.6M) and low complexity, ResNet50^42^ was preferred to other models. In the BACH (breast cancer histology images) competition, ResNet50, which adopts a highway network structure with shortcut connection, achieved a competitive performance^37^. Recently, another deep network, DenseNet^43^, use dense blocks to improve the feature redundancy and gradient vanishing problems. One study^44^ investigated the applicability of the DenseNet to the pathology image classification and segmentation for breast cancer. A DenseNet with 121 layers has approximately 8.1M parameters. Furthermore, MobileNet^45^ is a lightweight CNN architecture with fewer than 5M parameters and has achieved performance comparable to pathologists on the TMAZ dataset^46^. In general, these networks are significantly deep and complicated for optimizing and training.

Second, another potential limitation is reusability, since, for larger data sets, it may not be feasible to obtain detailed annotated regions of gigapixel images for training the model. In addition, computational pathology algorithms usually require precisely annotated tissue regions to train machine learning models and predict the slide label. In most real-world problems and for the task at hand, the ground-truth (GT) label for overall survival is generally provided at the patient level, and there are no detailed annotations about which regions of interest (ROI) from the tissue slides are more likely to impact survival. Among the existing approaches, the most common strategy is to select ROI of a WSI (mainly from tumor components) identified by a pathologist or a third-party application, and train a supervised learning model to predict survival. One important limitation is that such approaches introduce an inevitable bias to the model predictions and learn the prognostic features only from annotated ROIs. Therefore, the prognostic significance of the entire TME spatial organization remains largely unexplored. This leads to our hypothesis that machine learning models can be trained without region-level annotations and solely based on patient-level survival data to highlight the prognostic significance of different components of TME from H&E (Hematoxylin and Eosin) stained WSIs.

Our contributions are as follows:

1. Our framework demonstrates an objective and unbiased prognosis of using only primary tumor pathology images to predict survival time without prior knowledge of tissue composite structures. Indeed, our article used entire TME to predict the outcome of survival time using the cascaded learning framework. Taken together, increased evidence suggesting different components of the TME structure influence tumorigenicity and have prognostic potential in lung ADC.
2. We further investigate the effectiveness of the cascaded learning system SurBiRa (Survpatch model, Bin generation, and Random Forest algorithm) for the individual survival time prediction on the NLST and TCGA datasets, especially in the early-stage NLST (esNLST) and early-stage TCGA (esTCGA). Besides that, we separately reported the survival-time prediction of the framework by performing cross-validation on both datasets.

To our knowledge, this is the first study to explore the prognostic significance of the entire TME information on pathology images in individual survival time prediction with a cascaded learning framework. We believe that associated prior work and our present work will complement each other to ultimately improve prognostic accuracy.

## Results

### Study design

**Fig. 1:**
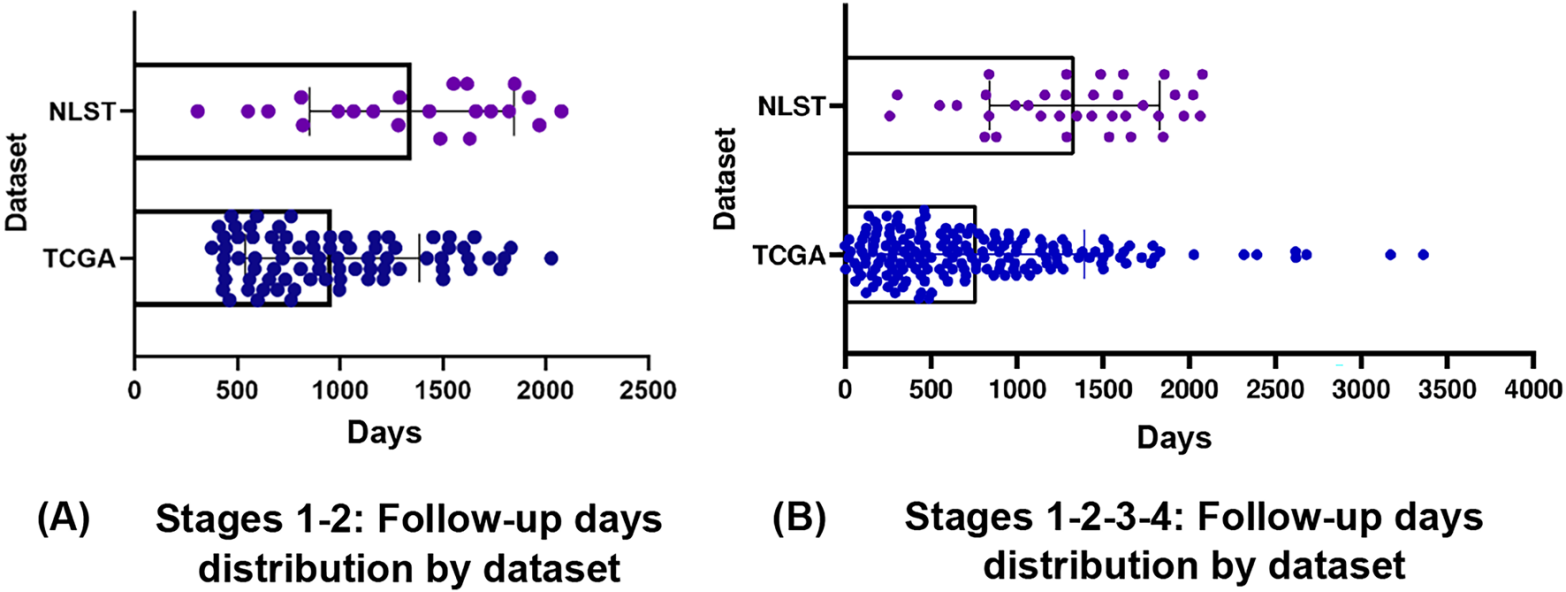
Follow-up days distribution of two datasets (NLST and TCGA) with different stages: (**a**) Stages 1-2, (**b**) Stages 1-2-3-4.

To develop and evaluate our cascaded learning framework to predict survival time among patients with ADC lung cancer, we collected pathology images from all dead patients in NLST and TCGA resources with a diagnosis of lung adenocarcinoma. Both data sets comprised patients with clinical characteristics and demographic data for each stage of cancer (from 1 to 4) as shown in Supplementary Table S1. Besides that, Fig. 1 shows the follow-up frequency distributions of 4 subsets: stages 1-2 NLST cohort, stages 1-2 TCGA cohort, stages 1-2-3-4 NLST cohort and stages 1-2-3-4 TCGA cohort. The follow-up days distribution of all stages 1-2-3-4 TCGA cohort has more outliers than the stages 1-2 TCGA cohort. These outliers are abnormal observations that skew the data distribution, and arise due to inconsistent data entry, or erroneous observations.

In particular, the NLST included data from 36 dead patients with ADC, yielding 148 pathology images. NLST images were manually classified by our pathologist from the Department of Pathology, Chonnam National University Medical School, into three main types (normal lung tissue and non-neoplastic, lymph node, and primary tumor) as shown in Supplementary Fig. S1. In detail, there are 48 normal lung tissue and non-neoplastic samples with 27 lymph node samples along with 73 primary tumor samples. Among the three main types, we first chose the best type for survival time prediction and then applied it to the associated problem. Especially, we conducted two groups: Primary Type (PT) and Whole Type (WT). The PT group contained only 73 primary tumor samples, while the WT group consisted of 148 samples including normal lung tissue and non-neoplastic, lymph node and primary tumor samples. Given the better results with the PT group, we selected primary tumor pathology images from both NLST and TCGA for further experiments.

We next investigate the effectiveness of the SurBiRa framework for the individual survival time prediction on NLST and TCGA cohorts as follows.

In the NLST dataset, we extracted 41,538 patches with a size of 512x512x3 at 20x magnification after removing regions that did not contain tissue. All patches belong to the TME region. Further, we randomly split the data 5-fold, four for training, and one for testing. We then performed cross-validation by switching the training and test datasets, ensuring that every patient’s is used once in the test set. Besides that, survival time (days) was used as a prognostic endpoint for this study. Finally, we compared the survival time prediction between early-stage (stages 1 and 2) and full dataset (stages 1, 2, 3 and 4).

In the TCGA dataset, there are 348 pathology images of 174 dead ADC patients. Particularly, there is a total of 57,664 patches with a size of 512x512x3 at 20x magnification extracted from the 348 pathology images. Similar to the experiments with the NLST, we performed a 5-fold cross-validation to evaluate the performance of our method. Then we obtained the results on independent cohorts: esNLST and esTCGA.

### Affection of primary tumor type to individual survival time prediction

One hypothesis was that pathology images obtained from different locations, such as the primary tumor region or other regions of lymph node metastases, would have different effects on the survival outcome efficiency. Therefore, we established an experiment for comparing the significant prognosis of PT group and WT group.

**Fig. 2:**
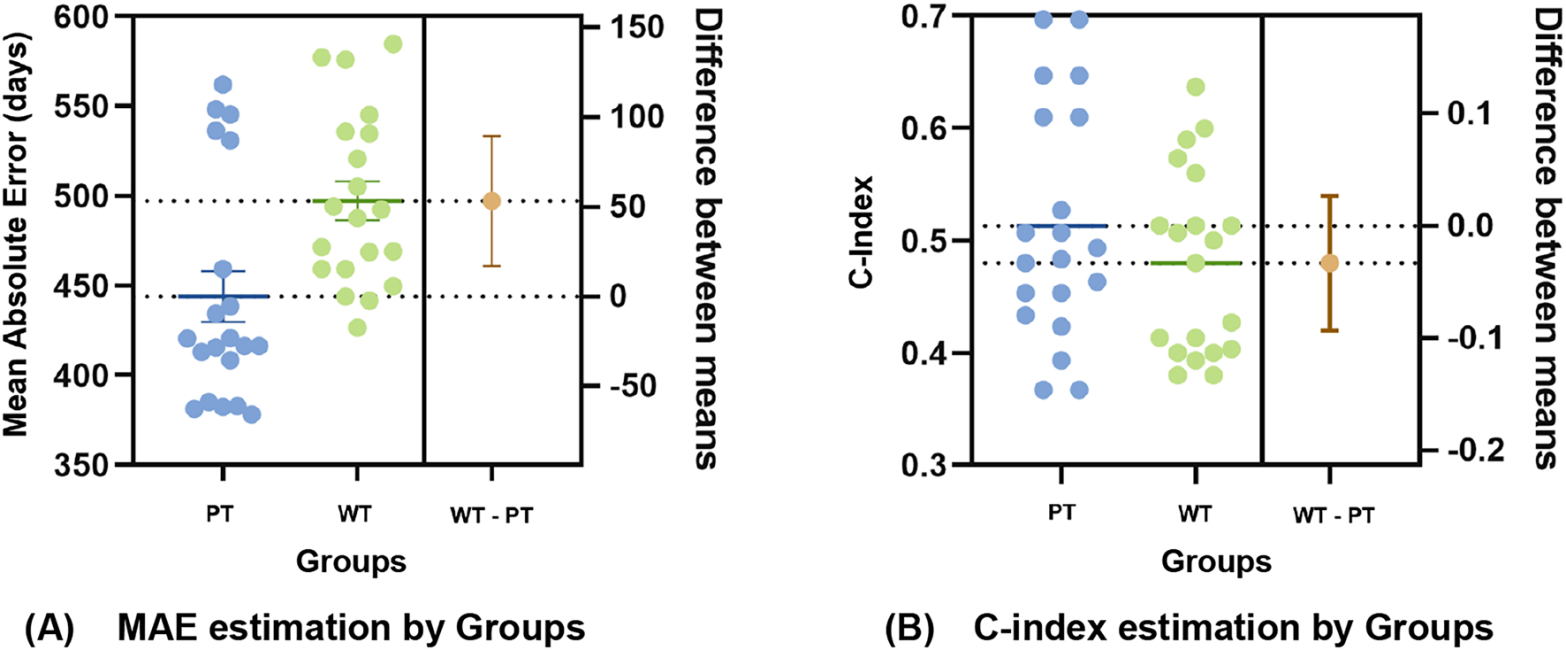
Comparison of results between the primary types (PT) group and the whole types (WT) group according to (**a**) Mean absolute error and (**b**) Concordance index.

As previously described, this experiment includes 148 pathology images in the NLST cohort. Our pathologist manually classified these samples into 73 primary tumor samples, 48 normal lung tissue and non-neoplastic samples, and 27 lymph node samples. Each classified pathology image was extracted into smaller patches. After patch extraction, each patch was assigned a survival label by assigning the slide-level survival time label to all patches. The patch-level prediction was passed through a sequence composed of Suvrpatch model, bin generation, and random forest regression to produce a WSI-level prediction. In this way, the effectiveness of the SurBiRa framework on primary tumor images group was investigated.

Accordingly, Fig. 2 shows the performance for the two comparisons between the PT group (73 primary tumor samples) and the WT group (73 primary tumor samples, 48 normal lung tissue and non-neoplastic samples, and 27 lymph node samples). Overall, the method that used only primary tumor types performed significantly better than that which used whole types, including primary tumor, lymph node, normal lung, and non-neoplastic. In other words, adding lymph node samples, normal lung tissue and non-neoplastic samples to primary tumor samples reduces the performance of the survival time prediction model. Notably, using only primary tumor images, the best MAE achieved an overall MAE score of 381 days with a C-index of 61.33%. Besides that, the difference between the MAE means of the two groups (PT and WT) is greater than 50 days (Fig. 2.a). Thus, using only pathology images from primary tumor regions decreased the MAE by more than 50 days compared to the WT group. In addition, the results of the C-index are further analyzed to compare the performance of two groups (Fig. 2.b). The C-index of the PT group is also higher than that of the WT group by around 3.33% (Fig. 2.b). Taken together, these results show that using only primary tumor pathology images leads to significant performance improvements for the survival time prediction model.

### Result of individual survival time prediction in the esNLST and esTCGA cohorts

We next evaluated the effect of the SurBiRa framework on the individual survival time prediction task. This experiment includes esNLST image patches and esTCGA image patches. Accordingly, slide-level prediction was produced from patch-level prediction through the SurBiRa framework. Then the patient-level survival time prediction was conducted from slide-level prediction by using an averaging function, which is described in detail in the methods section.

In particular, Table 3 illustrates the individual survival time prediction results over five models (InceptionResNetV2^47^, ResNet50^42^, DenseNet121^43^, MobileNet^45^ and our proposed model). Overall, it is evident that our proposed model is the best performer for most measurements. Regarding the size and the number of model parameters, the Survpatch model is the most optimal architecture in all five models, with 0.164MB and 0.033M, respectively. In terms of two evaluation metrics, our proposed method was superior to other models, with an MAE of 361 days, a C-index of 0.7 in the esNLST cohort. Similarly, in the esTCGA cohort, the Survpatch model achieved the best C-index with 0.58. Although the MAE of Survpatch model was placed third, the difference accounted for 32 days of the first place. It is clear that our proposed model plays an important role in improving the accuracy of the individual survival time prediction problem.

**Table 3:** Results of individual survival time prediction in esNLST, esTCGA cohorts. Performance was presented in higher-level analysis using the model size (MB), number of parameter, MAE and C-index. (The lower Size MB, #param, and MAE indicate better performance, the higher C-index indicates better performance).

Datasets			esNLST		esTCGA	
Measures	Size (MB) $\downarrow$	#params $\downarrow$	MAE (days) $\downarrow$	C-index $\uparrow$	MAE (days) $\downarrow$	C-index $\uparrow$
InceptionResNetV2^47^	215	55.90M	492.53	0.55	393.48	0.55
ResNet50^42^	8	25.6M	463.45	0.57	384.94	0.52
DenseNet121^43^	33	8.1M	421.65	0.62	333.38	0.56
MobileNet^45^	16	4.3M	448.27	0.67	351.82	0.51
Survpatch	0.164	0.033M	361.90	0.70	365.67	0.58

### Result of individual survival time prediction on independence cohorts

**Table 4:** Result of individual survival time prediction in independence cohorts. (The lower MAE indicates better performance, the higher C-index indicates better performance).

Train data	Test data			
	esNLST (n=73)		esTCGA (n=348)	
	MAE (days) $\downarrow$	C-index $\uparrow$	MAE (days) $\downarrow$	C-index ?
esNLST (n=73)	361. 90	0.70	508.62	0.59
esTCGA (n=348)	445.28	0.61	365.67	0.58

To ensure that our model was never evaluated on dependent samples, we trained and tested on different cohorts within Table 4. Table 4 shows the results of the individual survival time prediction in two independent cohorts (esNLST and esTCGA) using two evaluation metrics (MAE and C-index). In general, training and testing on the same set achieved better performance than that on the different sets. On esNLST test data, the results obtained from esNLST train data (MAE of 361.90 days, C-index of 0.7) were better than those from esTCGA train data (MAE of 445.28 days, C-index of 0.61). Moreover, the MAE of the esTCGA test data figures for training in esTCGA was 365.67 days, as opposed to 508.62 days for training on esNLST. Notably, the difference in C-index on esTCGA test data was not significant (C-index of 0.59 with esNLST data and C-index of 0.58 with the esTCGA train data).

In this experiment, our prognostic analysis of pathology images was evaluated with two separate cohorts from various regions. This was important as it ensured that our model was never evaluated on dependent samples. Overall, our framework is generalizable and will serve as a prospective multicenter clinical trial.

### The effect of cancer stages on individual survival time prediction performances

**Fig. 3:**
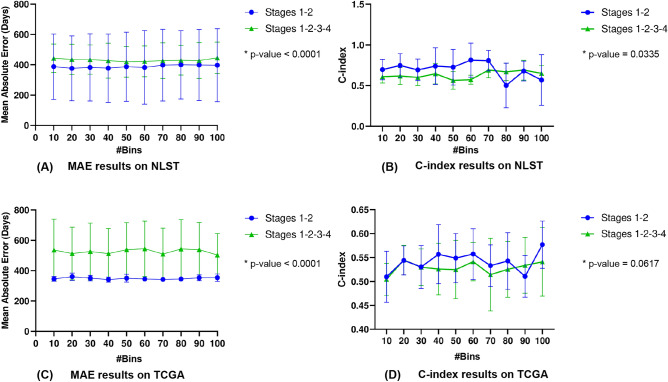
Comparison of survival time prediction performances using SurBiRa framework on two datasets: NLST and TCGA via two metrics: Mean Absolute Error and C-index with different stages: stages 1-2 and stages 1-2-3-4. (**a**) Comparison of MAE results on NLST. (**b**) Comparison of the C-index results on NLST. (**c**) Comparison of MAE results on TCGA. (**d**) Comparison of C-index results on TCGA. Lower MAE indicates better performance. Higher C-index indicates better performance.

Next, we took advantage of the SurBiRa framework to predict individual survival time on early-stage patients. Indeed, Fig. 3 shows a comparison of survival time prediction performances using the SurBiRa framework with the following parameters: 1) two cohorts (NLST and TCGA); 2) two metrics (MAE and C-index) and 3) different cases (stages 1-2 and stages 1-2-3-4). We used two-tailed p-values and $p-value<0.05$ was considered statistically significant. In general, the SurBiRa framework in the early-stage achieved better performance than all stages in terms of MAE in both cohorts ($p-value<0.0001$) as shown in Fig. 3.a and Fig. 3.c. Intuitively, our method may experience a favorable prognosis on early-stage patients compared with those in all stages, and our results show a comparable prognostic significance of the C-index (Fig. 3.b, $p-value= 0.0335$ and Fig. 3.d, $p-value= 0.0617$). It is encouraging that a SurBiRa framework shows prognostic significance in early-stage lung cancer.

### Ablation study

Lastly, we investigated the performance of each component in the SurBiRa framework. As shown in Supplementary Fig. S2, for the NLST cohort, the results show the difference for three combinations: (1) predictive model using the Survpatch model, (2) predictive model using the Survpatch model and Bin Generation method, (3) predictive model using SurBiRa framework: the Survpatch model, Bin Generation method and Random Forest regression. The survival time prediction results were observed with an increase in the C-index and a decrease in MAE for the Survpatch predictive model and the SurBiRa framework.

In addition, Supplementary Fig. S3.a and S3.b present the robustness evaluation of the C-index and MAE in the incorporation of Survpatch, Bin Generation and Random Forest on the esTCGA cohort. Unfortunately, Supplementary Fig. S3c and S3.d show a decrease in the C-index and an increase in MAE in the stages 1-2-3-4 TCGA cohort. This could potentially be explained by the fact that the training set of the stages 1-2-3-4 TCGA cohort has more outliers than stages 1-2 TCGA cohort (as shown in Fig. 1.b), which makes the C-index and MAE overfit on the test set.

## Discussion

In survival analysis, the censored patients are known (1) to be alive at some past follow-up but the investigator has since lost a trace of them, (2) to have changed hospital, (3) to have died of some cause unrelated to the disease. Therefore they did not suffer the outcome of interest. In another word, they are incomplete observations of the event time. This leads to our study which concentrates on regression tasks that must deal with uncensored patients where the patients’ actual time of death is observed.

Previous investigators have only focused on classifying survival outcome rather than predicting the individual survival time. We believe our pre-research study is the first to evaluate feasibility of the entire TME information on pathology images with the machine learning method for individual survival time prediction. In this study, we designed an automated workflow that identified thousands of patches from pathology images, then built and evaluated machine learning model to predict the individual survival time of patients with lung cancer. We also validated our methodology using pathology images from two independent datasets.

Additionally, the prognostic significance of tumor tissues, the stromal-tumor ratio, necrosis, cancer embolus, neural invasion, blood vessels, and inflammation has been identified through deep learning^48^. Furthermore, the prognostic significance of the features extracted from the original pathology image was presented in^10,49,50^. Similarly, our deep learning algorithm showed the diversity and heterogeneity of entire tissues on pathology images in prognosis, especially in the prediction of individual survival time.

Especially, a previous study for survival time analysis in the NLST dataset, tumor-only areas annotated by pathologists, achieved the C-index of 0.629 using the DeepConvSurv model (but no MAE value was reported). Since we trained the algorithm on whole tissues, the overall performance of survival time prediction was improved.

Furthermore, the use of more sophisticated networks (InceptionRestnetV2, Resnet, Densenet, etc.) in pathology images is time and memory consuming. Therefore, an advantage of our approach is that the Survpatch model uses well-customized parameters for image processing, saving time while improving performance. To our knowledge, this is the first study to show the utility of fully automated image features extracted from TME of pathology images to predict individual survival time.

Besides that, another advantage of our SurBiRa framework is its ability to improve performance on two inverse metrics. Particular, the C-Index and the MAE tend to pull in slightly different directions. Notably, our results demonstrated that the SurBiRa framework works effectively to increase the C-index value and decrease the MAE values. Note that this effect can change if there are outliers in the datasets. Outliers in the data may cause problems during model fitting. In addition, outliers could lead to less effective and less useful models. To ensure that the trained model generalizes well to the valid range of test input, it is important to detect and remove outliers before building an machine learning model.

Together, we proposed a survival time prediction algorithm in a cascaded framework from pathology images of Lung Adenocarcinoma. The algorithms successfully visualized the effect of the patch-based method on pathology images which was independent of other clinical variables. In addition, the performance of the proposed method was evaluated in terms of the MAE and the C-Index. Moreover, our presented results have several interesting aspects. Primarily, it overcomes the need for manual annotations of tumor regions, which is a laborious and time-consuming task. And secondly, we believe this is the first study to show the utility of cascaded learning framework to predict individual survival from only pathology images without tumor tissues ROIs, such as there is an increasing interest in consolidated prognostic significance of other tissues behind tumor issues.

Limitations: This study presents an emerging machine learning tool that can help optimize clinical trial design, but also comes with several limitations. The proposed SurBiRa framework only utilized pathological imaging information. In the future, clinical information (e.g., patient characteristics) as well as other domain knowledge (e.g., genomics) will be incorporated into the SurBiRa framework to further improve the performance. Secondly, stage 3-4 patients are diagnosed at a late time, resulting in a short time from detection to death. Meanwhile, the presentation of pathology images has a more severe prognosis, which reduces the efficiency of the model. Thirdly, we also found that several factors affect the performance of the framework as significant obstacles to prognosis, including poor staining of H&E, image opacity, non colorization, or poorly differentiated tumors. A standard procedure for the preparation of H&E slides should be established and distributed in the pathology image processing for a better application of AI-assisted prognosis. Finally, the pathology analysis is a time-consuming and expensive process. In the future, we will expand the datasets in order to strengthen the current research.

## Methods

This section provides an explanation of the materials and methods used to predict individual survival time. The model was trained on extracted patches from pathology images using ground truth survival time information provided at the patient level (for patient data that lacks precisely marked regions).

### Data pre-processing

**Fig. 4:**
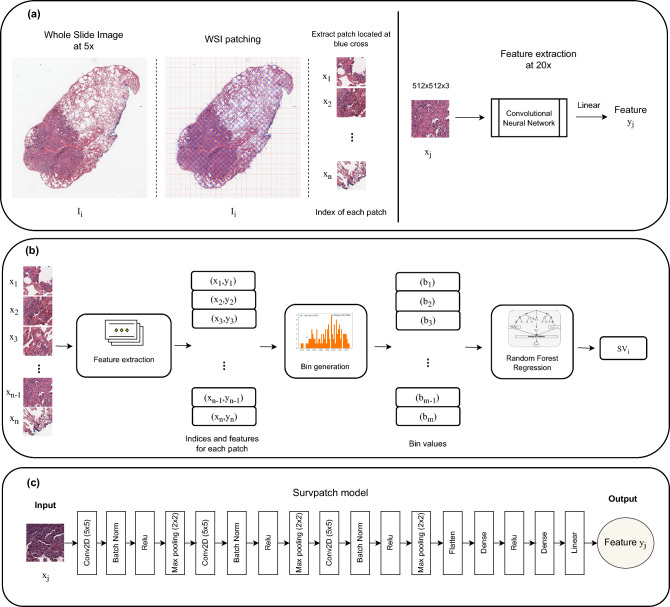
Schematic overview of the SurBiRa framework for individual survival time prediction of Lung Adenocarcinoma using pathology images. (**a**) Data pre-processing and feature extraction using a convolutional neural network. (**b**) The cascaded learning framework, SurBiRa, includes three steps: feature extraction using the Survpatch model, features normalization using the Bin generation method and prediction using the random forest regression method. (**c**) Our proposed model, Survpatch architecture.

Figure 4 shows a schematic illustration of the cascaded learning framework, and Fig. 4.a presents the pre-processing step. We processed and analyzed all our WSI data at 20$\times$ magnification. Due to the lack for labeled ROIs of the TME region and the intractable computational expense of deploying a convolutional neural network (CNN) directly to the entire spatial extent of each WSI, we utilized the patch-based method. Under the patch-based method, each pathology image was extracted to smaller regions, enabling the CNN model to learn directly from the patch level during training. Notably, we divided the tissue regions in each WSI into Nx512x512x3 image patches, where N varies with the area of each slide. In addition, we used data augmentation as an effective way to enhance the sufficiency and diversity of training data. The data augmentations listed in this study are: random contrast, random gamma and random brightness.

### Workflow for survival time prediction by using deep learning - SurBiRa framework

For survival time regression, we considered four convolutional neural network (CNN) models: InceptionResNetV2^47^, ResNet50^42^, DenseNet121^43^ and MobileNet^45^. Then we optimized our proposed model.

Due to high magnification and large datasets, patch-based deep learning models (e.g., InceptionV2, InceptionResNetV2, etc.), have been applied recently to pathology image analysis with effective classification results. However, the training process is exhaustive and time-consuming. Indeed, the models illustrated in Table 3 use a larger sample size and more parameters than our proposed model. In our study, we use the new CNN architecture (Survpatch model) to predict the survival times of patients from their pathology images.

Regarding Survpatch model, our cascaded learning framework named SurBiRa has three main components as shown in Fig. 4.b, including a) a CNN - Survpatch model, b) the Bin generation method, and c) the Random Forest regression method.

In the practical application scenario, it is assumed that patient *P* has *z* pathology images ( $I_1$, $I_2$,..., $I_z$ ). Each patient has a varied number of pathology images.

Given the observations $(P,I_{(i \ldots z)},t),i=1,2,\ldots ,z$, *P* is an individual patient, $I_{i}$ is the pathology image of patient, *z* is the number of pathology images of each patient *P*, and *t* equals the observed time to the event of interest for each *i*. Each pathology $I_{i}$ is extracted to patches ($x_{1},x_{2},\ldots ,x_{n}$) and then inputted into the Survpatch model.

Our proposed model is named the Survpatch model, and it is shown in Fig. 4.c. Particularly, the Survpatch model inputs a patch and predicts the regression confidence of a patch. Then, the output and ground-truth values are used to calculate the loss value to update the weights in each hidden layer of the model.

The output of the Survpatch model follows equation (1):

1 $$\begin{aligned} y_i=f(c+ \sum _{i=1}^n(x_i \times w_i )) \end{aligned}$$

where the product of patch input ($x_1,x_2,\ldots ,x_n$) and corresponding weights ($w_1,w,\ldots ,w_n$) is added to a bias (*c*) and inputted into the activation function f applied to the weighted sum of the inputs.

However, the number of patches differs among pathology images based on their varied sizes. Therefore, we conducted ablation Bin generation. The purpose of the Bin generation step was to reduce the number of features, by combining neighboring values into a single value that could represent single feature values.

Let *n* be defined for the number of patches ($x_1,x_2,\ldots ,x_n$) per each pathology image $I_i$. Let *max* and *min* be maximum and minimum values in a range of patches ($y_1,y_2,\ldots ,y_n$). Let *IQR* be the interquartile range of ($y_1,y_2,\ldots ,y_n$). The best bin value is selected by Freedman–Diaconis^51^ rule as illustrated in equation (2).

2 $$\begin{aligned} m = \root 3 \of {n} \frac{max-min}{2 \times IQR(x) } \end{aligned}$$

At the inference time, our objective was to predict the survival time of each patient. However, our trained Survpatch predicts patches, not entire slides, and the varying number of pathology images for each patient also represents a complication as well. Therefore, we first applied Random Forest over the normalized features on the entire image to obtain the survival time of each pathology image. We then calculated the individual survival time of each patient based on the average of all predicted pathology image values.

Given an input $(b_i,t_i)_{i=1}^m$, the survival time of each pathology image *I* is calculated as equation (3).

3 $$\begin{aligned} SV_i= \frac{1}{m} \sum _{j=1}^m w_{b_i,y'}^j \times t_i \end{aligned}$$

where *m* is a number of bins, *b* is the bin value, $y'$ is the predicted value, *t* is the observed time to the event of interest, and *W* is the weight of each node in the random forest model.

Next, due to the different number of images for each patient, we evaluated the performance of our method via equation (4).

4 $$\begin{aligned} SV_p= \frac{1}{p} \sum _{i=1}^pSV_i \end{aligned}$$

$SV_p$ defines Survival Time per patient *P*. In addition, *p* is the number of pathology images of each patient and $SV_i$ is the survival day corresponding to each pathology image.

Accordingly, a Survpatch model was trained for fifty epochs with Adam optimization, beginning with an initial learning rate of 0.001 and decaying by a factor of 0.9 every epoch. The proposed model used the Linear activation function for the output layer and the mean absolute error loss function. Images were normalized from 0 to 1 value for each RGB (red, green, blue) channel. Python (version 3.6) and Python libraries (Keras, version 2.1.5; open slide-python, version 1.1.1; and Tensorflow-gpu, version 1.8.0) were used. In the Bin generation step, function binned_statistic of scipy.stats library was used to compute a binned statistic for all extracted features from the Survpatch model with a statistic as mean and bin number calculated by equation (2). In the Randomforest regression step, a RandomForestRegressor function of sklearn.ensemble library was used with the number of trees in the forest is 10, and the function to measure the quality of a split is mean absolute error.

### Performance evaluation

Here, the definition of an event was limited to the death of the same lung ADC patient. Therefore, we used the mean absolute error (MAE) and the concordance index (C-index) as metrics for assessing the predictive performance. Normally, MAE^52,53^ is the measure of errors between the truth values and the predicted value, and it was used in the Python programming language throughout this paper. Besides that, the concordance index (C-index)^48,54^ is the frequency of concordant pairs among all pairs of subjects. For these experiments, this metric was calculated using R version 20.4. In addition, a two-sided $p-value < 0.05$ was considered statistically significant.

## Conclusions

The present study examined the ML-based survival time predictive model of patients with early-stage lung adenocarcinoma and evaluated the TME that contributes to survival outcomes prediction. This suggests that the utility of the ML-based cascaded learning framework to predict individual survival time from whole TME in pathology images. The prognostic significance of other tissues behind tumor issues may be necessary to improve the performance of survival outcome prediction. These findings provide valuable insights into the TME that influence survival outcomes prediction in patients with lung adenocarcinoma by using pathology images and machine learning methods. In the future, we will investigate the prognostic significance of each tissue type for survival outcome prediction.

## Supplementary Information

Supplementary Information.

## Data Availability

The datasets that support the findings of this study are available online in NLST (https://cdas.cancer.gov/nlst/) and The Cancer Genome Atlas Lung Adenocarcinoma (TCGA-LUAD, https://wiki.cancerimagingarchive.net/display/Public/TCGA-LUAD).
