# Supplementary material for: Pathology image-based predictive model for individual survival time of early-stage lung adenocarcinoma patients
Source: Sci Rep. 2025 Oct 15;15:35964. doi: 10.1038/s41598-025-16073-7 (PMC12528468; doi:10.1038/s41598-025-16073-7)
Supplement: Supplementary file 1 — Supplementary Information. [file 41598_2025_16073_MOESM1_ESM.pdf]

Pathology Image-Based Predictive Model for Individual Survival Time of Early-Stage Lung Adenocarcinoma Patients

Vi Thi-Tuong Vo[1], Hyung-Jeong Yang[1], Taebum Lee[2, *], and Soo-Hyung Kim[1,*]

[1]Department of Artificial Intelligence Convergence, Chonnam National University, Gwangju, 61186, South Korea
[2]Department of Pathology, Chonnam National University Medical School, Gwangju, 61186, South Korea
[*]dr.taebum@gmail.com, shkim@jnu.ac.kr

Table

Table S1. A concise summary of clinical characteristics for NLST and TCGA datasets.

Patient characteristics	NLST dataset	TCGA dataset
Number of patients	36	174
Total pathology images (n)	148	348
Total patches (p)	41538	57664
Age	64.60 ± 5.57	65.77 ± 10.59
Gender (Male/Female)	55.56 % / 44.44%	48.28% / 51.72%
**Stages**		
Stage 1	15	61
Stage 2	7	52
Stage 3	11	46
Stage 4	3	15
Median follow-up time (days)	1327.40	764.85

Figures

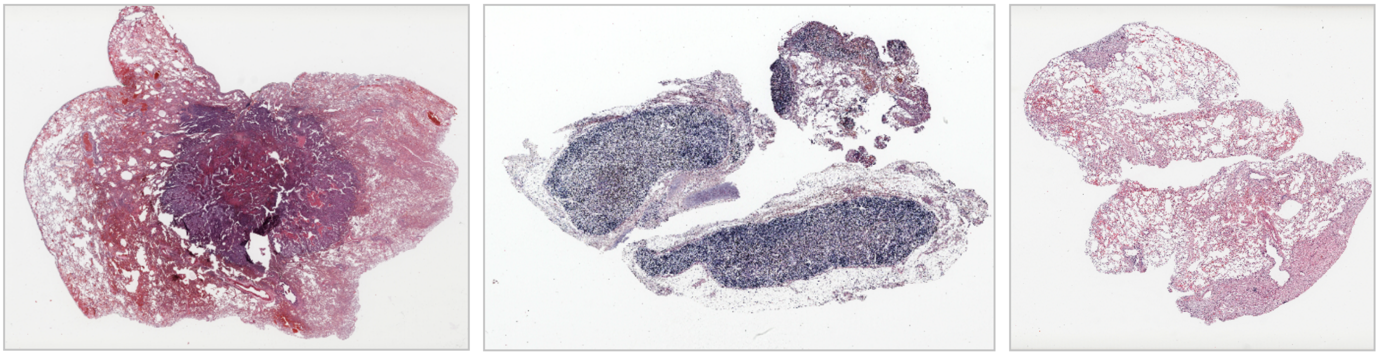

(a) Primary tumor

(b) Lymph node

(c) Normal lung tissue and non-neoplastic

Figure S1. Examples of pathology image types. (a) Primary tumor sample, (b) Lymph node sample, (c) Normal lung tissue and non-neoplastic sample

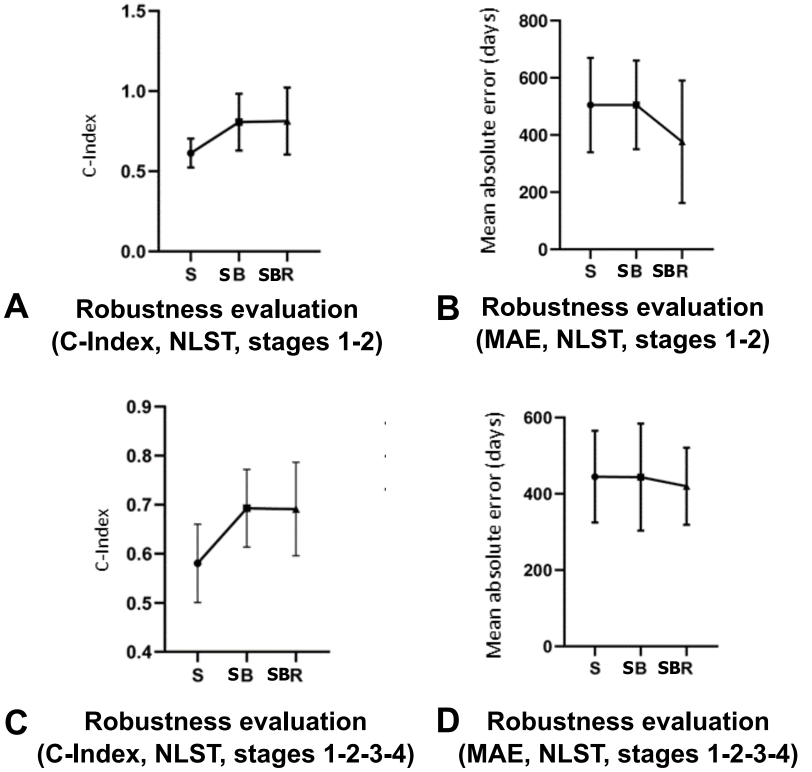

- **S**: Survpatch - **SB**: Survpatch + Bin Generation - **SBR**: Survpatch + Bin Generation + Random Forest

Figure S2. Assessment of the direction of the C-index and MAE results according to three combinations: (1) Only Survpatch, (2) Survpatch + Bin Generation, (3) Survpatch + Bin Generation + Regression Random Forest on the NLST cohort. (a) Experiment results on stages 1-2 NLST cohort through C-index. (b) Results of the experiment in stages 1-2 NLST cohort through MAE. (c) Results of the experiment in stages 1-2-3-4 NLST cohort through C-index. (d) Results of the experiment in stages 1-2-3-4 NLST cohort through MAE. S defines for Survpatch model, B defines for Bin generation and R defines for Random Forest algorithm. (Lower MAE indicates better performance, higher C-index indicates better performance)

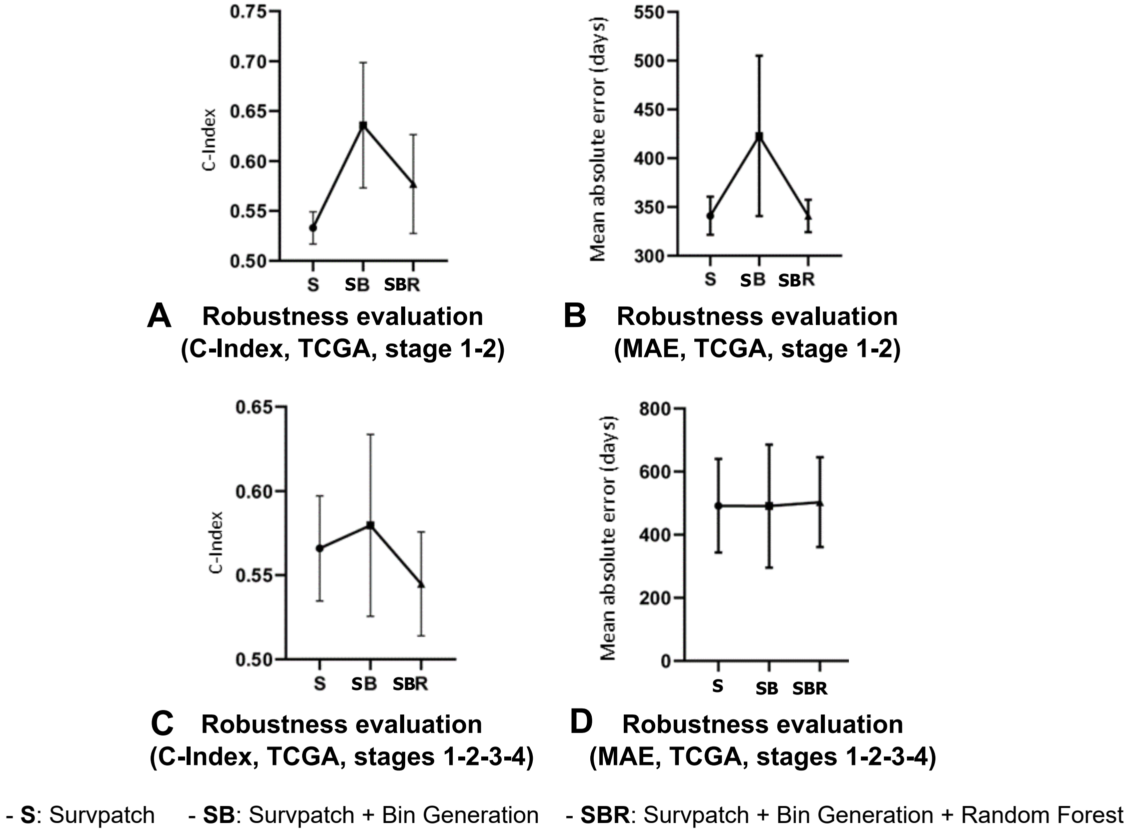

Figure S3. Assessment of the direction of the C-index and MAE results according to three combinations: (1) Only Survpatch, (2) Survpatch + Bin Generation, (3) Survpatch + Bin Generation + Regression Random Forest on the TCGA cohort. (a) Results of the experiment in stages 1-2 TCGA cohort through the C-index, (b) Results of the experiment in stages 1-2 TCGA cohort through MAE. (c) Results of the experiment in stages 1-2-3-4 TCGA cohort through C-index. (d) Results of the experiment in stages 1-2-3-4 TCGA cohort through MAE. S defines the Survpatch model, B defines the Bin generation and R defines the Random Forest algorithm. (Lower MAE indicates better performance, higher C-index indicates better performance).
